# ENDOSCOPIC STENT FOR TREATMENT OF ESOPHAGOJEJUNOSTOMY FISTULA

**DOI:** 10.1590/S0102-67202015000300018

**Published:** 2015

**Authors:** Marcus Fernando Kodama Pertille RAMOS, Bruno da Costa MARTINS, Aline Marcilio ALVES, Fauze MALUF-FILHO, Ulysses RIBEIRO-JÚNIOR, Bruno ZILBERSTEIN, Ivan CECCONELLO

**Affiliations:** Cancer Institute, Hospital das Clínicas, University of São Paulo Medical School, São Paulo, Brazil.

## INTRODUCTION

Fistula from esophagojejunostomy is still one of the most feared complications after total gastrectomy. Despite the development of new surgical devices and techniques, it remains a major concern with an incidence around 5%^10^. The use of endoscopic stents has brought the possibility of another form of fistula treatment^3^. However, this technique hasn´t yet been fully incorporated into clinical practice. This article describes a case of an esophagojejunostomy fistula that was successfully treated with an endoscopic stent. A literature review about this issue also follows.

## CASE REPORT

**FIGURE 1:**
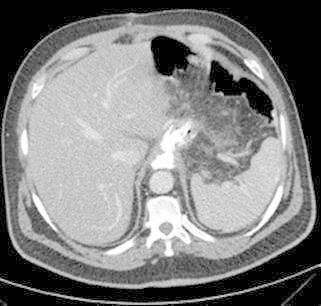
Abdominal CT-Scan with contrast leak

A 61- year-old male patient was diagnosed with an infiltrative 6 cm tumor located in the lesser curvature of the medium gastric body invading cardia. The biopsy revealed a diffuse adenocarcinoma with signet-ring cells. Co-morbidities included morbid obesity (BMI 40.8) and arterial hypertension. Staging CT-scan showed a thickening of the gastric lesser curvature without any lymph node enlargement. The patient underwent total gastrectomy with D2 lymph node dissection and Roux-en-Y reconstruction. Esophagojejunal anastomosis was performed with a 25 mm circular stapler with intact resection rings. No leakage occurred after methylene blue testing. The anastomosis was drained with bilateral tubular silicon drains. On the 5^th^ postoperative day, the patient presented diffuse abdominal pain and drainage of enteric fluid in the tubular abdominal drain. A CT-Scan with oral contrast demonstrated a leakage in the anastomotic area as shown in Figure 1.

Since it was an early fistula associated with peritonitis, an exploratory laparotomy was performed and revealed a suture dehiscence of 40% of the posterior wall of the esophagojejunal anastomosis. No specific local factors were noted that could explain the early occurrence of the fistula. Latter, the patient confessed unauthorized drinking of liquids since the first postoperative day. A suture of the dehiscence area was performed along with a nutritional jejunostomy, nasoenteric tube for decompression, and drainage of the cavity. Two days after the revisional surgery, leakage of enteric liquid in the abdominal drain occurred again, but without clinical signs of peritonitis. After discussion and evaluation of the patient clinical status, it was decided for a non-surgical treatment of this recurrent fistula. Patient remained stable with antibiotics, parenteral and enteral nutrition. 

**FIGURE 2:**
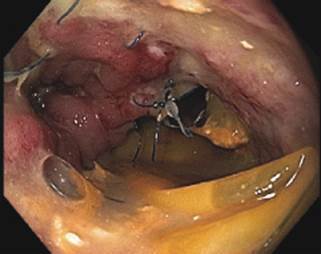
Endoscopy showing fistula

An upper digestive endoscopy was performed seven days after this new leakage to evaluate the anastomosis and for planning a possible endoscopic treatment. The endoscopy showed a dehiscence of 50% of the posterior wall of the anastomosis and no signs of obstruction in the jejunal loop beyond the anastomosis (Figure 2).

**FIGURE 3:**
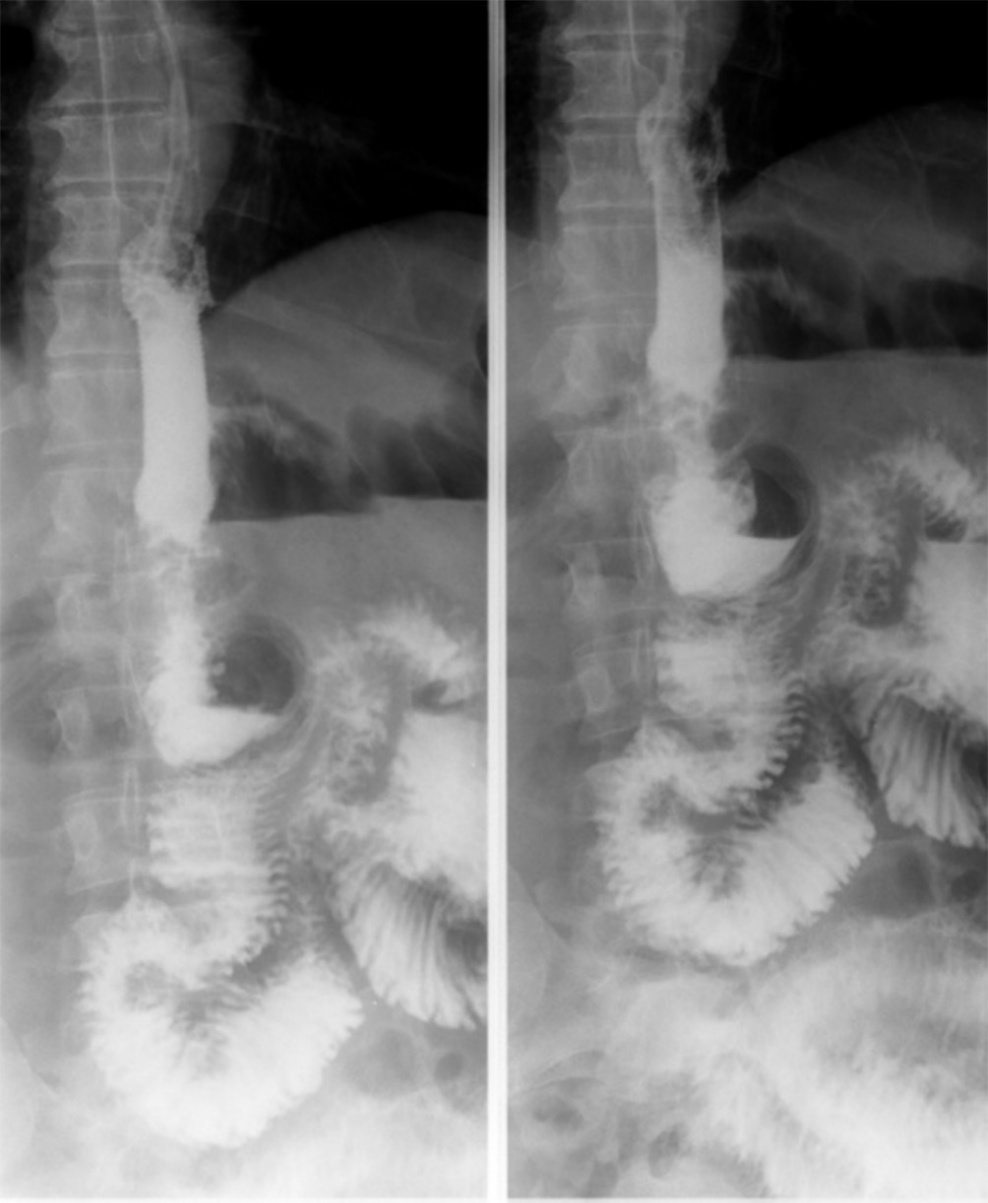
Esophagography five days after stent placement showed complete occlusion of the leakage

A fully covered metal stent was placed occluding the anastomotic leakage. To prevent stent migration, an external anchoring was performed with a piece of dental floss passed through the upper flange of the stent, as previous described by our group^2^. Since the stent is fully covered, a pediatric forceps was used to puncture the sheath, allowing the passage of the dental floss through the mesh. An esophagography with iodine dye taken the next day still revealed that there was a small leakage. Another esophagography, five days after the placement of the stent, didn´t reveal any leakage (Figure 3) and oral liquid intake was restarted. 

The abdominal drainage diminished dramatically without any more episodes of enteric liquid. The patient was discharged from the Hospital one week after the stent placement, with a closed jejunostomy and a strictly soft oral diet. The stent was easily removed by endoscopy five weeks later and no signs of fistula were seen at this time. 

## DISCUSSION

Despite the decreasing incidence of gastric cancer in Brazil, statistics show that of those that are diagnosed, there has been an increasing incidence of proximal lesions resulting in a higher proportion of total gastrectomies compared to subtotal ones^6^. The use of perioperatory chemotherapy as well as salvage and palliative surgery has extended the indications of surgical resection. This trend has pushed surgeons to challenge themselves by performing operations on more critical patients with higher risks of complications including fistula. At our Institute, after 169 consecutives esophagojejunostomies for total gastrectomies between 2009 and 2014 there were nine cases (5,3%) of fistula with three fatal outcomes. Specifically, when degastrectomy was performed the incidence of fistula was higher occurring in four out of 30 cases (13,3%). 

Conservative treatments with antibiotics and oral fasting have been used in patients who have oriented and drained fistulas with no clinical symptoms. Cases of peritonitis require a surgical approach with suture of the dehiscence, a new anastomosis or even an esophagostomy. Another aspect is timing of fistula occurrence. Early fistula suggests a technical failure when performing the anastomosis and should be corrected with a new surgical approach. An early fistula has the tendency to develop peritonitis since an inflammatory blocking isn´t fully formed.

Recently^5^, temporary endoscopic stent placement has emerged as a minimally invasive treatment option for benign esophageal ruptures and leaks. A randomized prospective study comparing endotherapy with surgery hasn't been found in today's literature but favorable outcomes with low morbidity and mortality were reported from several series with stent placement^1^ ^4^ ^5^ ^7^ ^8^ including a recently published large review^3^. Before stent placement it is very important to guarantee an adequate drainage of fluid collections. Once drainage is performed, stents can seal the leaks and offer protection of the mucosal wall. 

The extension of dehiscence must be considered before the stent placement. Fistulas with a dehiscence area inferior to 50 % of the anastomosis have good results with stent. On the other hand, if the dehiscence area is superior to 50% the possibility of fistula closure is lower and it may reflect a major surgical technical problem, such as ischemia and tension. Postoperative time of fistula appearance is also an important factor. As mentioned before, early fistula lacks adequate inflammatory blocking, bringing the risk of a complete anastomosis rupture after the stent deployment. Placement of the stent after the 7^th^ postoperative day, when the inflammatory blocking around the anastomosis is more consolidated is considered safer. An esophagography with iodine dye should be done after stent placement and, if leakage occlusion is confirmed, oral intake may be resumed while tissue healing takes place. 

There are three types of commonly used stents: partially covered self-expandable metal stent (PSEMS), fully covered self-expandable metal stent (FSEMS) and self expanding plastic stent (SEPS). Clinical success has been very similar among studies comparing the different types of stents, without clear benefit of one type over another (PSEMS: 48%-81%, FSEMS: 48%-90% and SEPS 67%-100%)^1^ ^4^ ^5^ ^7^ ^8^. Van Boeckel et al^8^ compared the outcomes of three different stents designs in the treatment of benign esophageal rupture or anastomotic leakage. Fifty-two patients were treated either with a FSEMS, PSEMS or SEPS. Endoscopic stent removal was successful in all but eight patients treated with a PSEMS due to tissue ingrowth. Clinical success was achieved in 76% (PSEMS: 73%, FSEMS: 83%, SEPS: 83%) after a median stenting time of 39 days (range 7-120). Twenty-four patients had complications, including: tissue in- or overgrowth (n=8), stent migration (n=10), ruptured stent cover (all PSEMS; n=6), food obstruction (n=3), severe pain (n=2), esophageal rupture (n=2) and hemorrhage (n=2). One patient died of a stent-related cause. 

When choosing the type of stent, the endoscopist must be aware of the pitfalls for each stent. Partially covered metal stents (PCMS) cause tissue ingrowth as early as one week after placement^8^, impairing its removal with risks of bleeding and perforation. In a recent study, all four patients who were treated with a PCMS for benign esophageal rupture suffered perforation when stent removal was attempted^5^. On the other hand, fully covered stents (either metallic or plastic) are more prone to migration (20-42%)^1,^ due to its reduced anchoring capacity^9^. However, migration can be minimized with some endoscopic techniques, such as clipping the proximal edge of the stent^9^ or external fixation. In this related case and in a similar case after, we had the opportunity to place an endoscopic fully covered stent with external dental floss fixation with adequate closure of the fistula and a successful removal of the stent after five weeks.
